# Speed versus endurance tradeoff in plants: Leaves with higher photosynthetic rates show stronger seasonal declines

**DOI:** 10.1038/srep42085

**Published:** 2017-02-10

**Authors:** Yong-Jiang Zhang, Lawren Sack, Kun-Fang Cao, Xue-Mei Wei, Nan Li

**Affiliations:** 1 Key Laboratory of Tropical Forest Ecology, Xishuangbanna Tropical Botanical Garden, Chinese Academy of Sciences, Mengla, Yunnan 666303, China; 2 Department of Organismic and Evolutionary Biology, Harvard University, Cambridge, MA 02138, USA; 3 School of Biology and Ecology, University of Maine, Orono, ME 04469, USA; 4 Department of Ecology and Evolutionary Biology, University of California, 621 Charles E. Young Drive South, Los Angeles, CA 90095 1606, USA; 5 Plant Ecophysiology and Evolution Group, State Key Laboratory for Conservation and Utilization of Subtropical Agro-bioresources, Guangxi Key Laboratory of Forest Ecology and Conservation, College of Forestry, Guangxi University, Nanning, Guangxi 530004, China; 6 National Cycad Germplasm Conservation Center, Fairylake Botanical Garden, Shenzhen and Chinese Academy of Sciences, 160 Xianhu Rd., Liantang, Shenzhen 518004, China

## Abstract

We tested for a tradeoff across species between plant maximum photosynthetic rate and the ability to maintain photosynthesis under adverse conditions in the unfavorable season. Such a trade-off would be consistent with the observed trade-off between maximum speed and endurance in athletes and some animals that has been explained by cost-benefit theory. This trend would have importance for the general understanding of leaf design, and would simplify models of annual leaf carbon relations. We tested for such a trade-off using a database analysis across vascular plants and using an experimental approach for 29 cycad species, representing an ancient plant lineage with diversified evergreen leaves. In both tests, a higher photosynthetic rate per mass or per area in the favorable season was associated with a stronger absolute or percent decline in the unfavorable season. We resolved a possible mechanism based on biomechanics and nitrogen allocation; cycads with high leaf toughness (leaf mass per area) and higher investment in leaf construction than in physiological function (C/N ratio) tended to have lower warm season photosynthesis but less depression in the cool season. We propose that this trade-off, consistent with cost-benefit theory, represents a significant physio-phenological constraint on the diversity and seasonal dynamics of photosynthetic rate.

Photosynthetic capacity is the fundamental determinant of plant carbon gain and growth, and in most species shows pronounced seasonal dynamics, declining in the dry season or cool season. Further, the degree of seasonal decline in photosynthetic rate is highly variable among species within and across biomes^1^,^2^. Many studies of the seasonal dynamics of photosynthesis have related variation to xylem water transport, enzyme activity, mesophyll conductance, stomatal conductance, and electron transport efficiency^2^,^3^,^4^,^5^. However, studies of general leaf design principles or tradeoffs that regulate the seasonal dynamics of photosynthesis have been lacking though they will be fundamental for predicting the response of plants to a future with increased climate extremes. Here we test a novel hypothesis for a tradeoff between photosynthetic capacity and its maintenance into unfavorable conditions.

A tradeoff between maximum performance (*e.g.*, speed) and endurance is found in athletes^6^ and some animals^7^,^8^. Similarly, a tradeoff between leaf photosynthetic capacity (performance) and its maintenance in adverse seasonal conditions (endurance) would be expected based on cost-benefit theory^9^. Indeed, an analogous trade-off is well-recognized between leaf photosynthetic capacity and leaf longevity across diverse species and plant communities worldwide^10^,^11^,^12^. According to a now-classic analysis^9^, a trade-off between maximum photosynthetic performance under high resource conditions and the ability to maintain performance under low resource conditions would lead to variation in leaf longevity, such that leaves with higher performance under high resource conditions would have shorter lifetimes than those able to maintain performance under lower resource conditions, and which therefore invested in cell and tissue-level mechanisms to support an extended lifetime. Indeed, across species of angiosperms and gymnosperms, tough leaves adapted to resist environmental stress and herbivores have higher investment in leaf construction, i.e., in leaf mass per area (LMA), typically reflecting a thicker leaf with higher fiber content, greater mass density, low N concentration, and higher N allocation to cell wall^13^,^14^. These modifications tend to increase leaf longevity but result in low maximum photosynthetic rates per leaf area and per mass because of lower total concentration of N on an area and mass basis, lower allocation of N to photosynthetic machinery, and thicker cell walls resulting in higher diffusion resistance to CO_2_ to the chloroplasts^11^,^15^,^16^,^17^,^18^. Precisely the same logic would lead to the expectation of a negative relationship between maximum photosynthetic rate in the favorable season and the ability to maintain photosynthetic rate in the unfavorable season, a pattern that would have important implications, though this has not previously been tested to our knowledge.

Here we test this hypothesis first using published data for 36 evergreen angiosperm and conifer species from 22 studies across the temperate zone (Table S1). Second, we conducted a common garden experiment to minimize the influence of diverse growing conditions with 29 cycad species in two gardens with a pronounced cool season and substantial chilling temperatures (Fig. S1, Materials and Methods): Xishuangbanna Tropical Botanical Garden (XTBG) and Fairylake Botanical Garden (FBG), Southern China (Table S2). Cycads represent a useful platform for testing the generality of leaf trait relationships especially as they have been found to converge with angiosperms in leaf design relationships, such as the relationship of photosynthetic rate and nutrient concentrations with LMA^19^. Notably, a tradeoff between photosynthetic capacity and the maintenance of photosynthesis in the unfavorable season might be difficult to resolve across species with relatively short leaf lifespans if photosynthetic depression in the unfavorable season coincides with the effects of ontogeny on leaf structure and photosynthetic rate^20^. Cycads tend to have long leaf lifespans and a diversity of leaf morphology, structure, and photosynthetic capacity^19^,^21^, and thus represent an ideal system for testing this leaf design hypothesis.

## Results and Discussion

**Figure 1:**
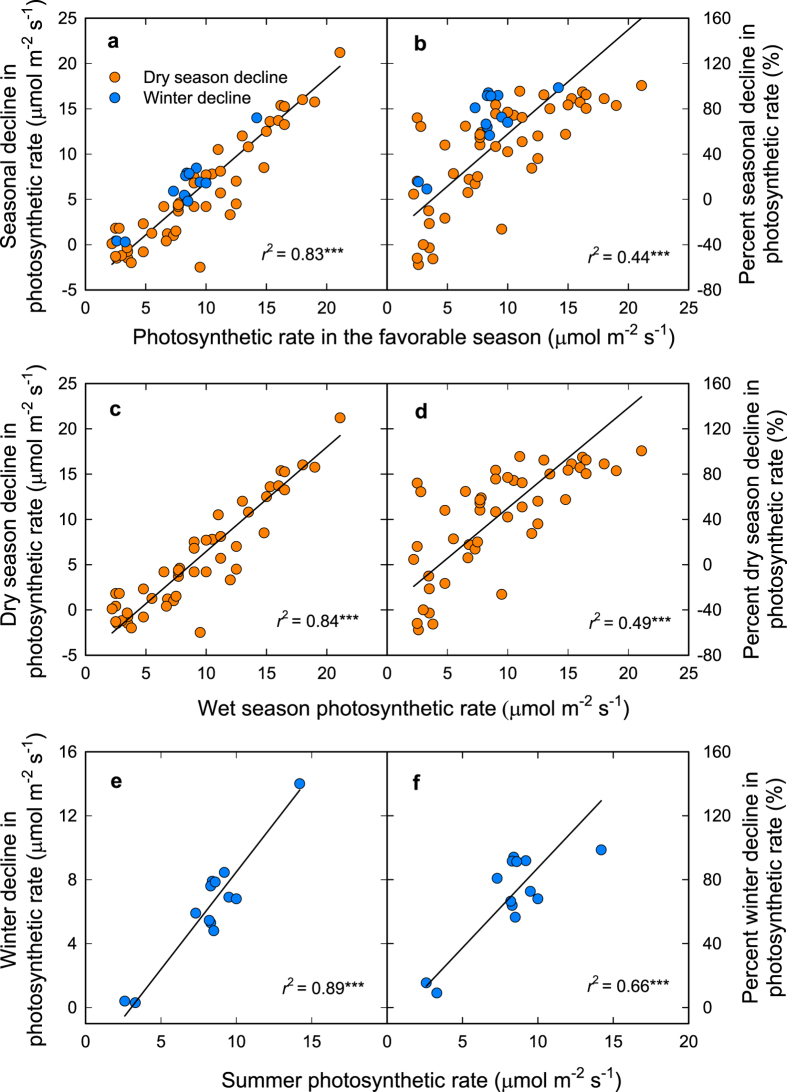
The relationship between maximum photosynthetic performance and seasonal maintenance in the unfavorable season in seed plants (compiled data). (**a**) Absolute or (**b**) relative (percent) seasonal decrease in net photosynthetic rates (*A*_a_) in relation to *A*_a_ in the relatively favorable season (Summer or wet season). Absolute (**c**) or relative (percent) (**d**) dry season decrease in *A*_a_ in relation to wet season/winter *A*_a_. Absolute (**e**) or relative (percent) (**f**) winter decrease in *A*_a_ in relation to summer *A*_a_. Data are compiled from the literature (Table S1). Lines are standardized major axis (SMA) lines fitted to the data.

We found strong support for the hypothesized tradeoff between maximum photosynthetic rate in the favorable season and the ability to maintain photosynthesis seasonally in both the analysis of published data for conifers and angiosperms and the experimental study on cycads. The analysis of published data for conifers and angiosperms showed that leaf light-saturated net photosynthetic rate per leaf area (*A*_a_) in the favorable season was positively correlated with the absolute or relative (percentage) decline of leaf light-saturated net photosynthetic rate per leaf area (*A*_a_) in the unfavorable season (Fig. 1a,b). The relationships remained when plants were separated into the groups experiencing photosynthetic decline due to summer drought or winter cold (Fig. 1c,d,e,f).

**Figure 2:**
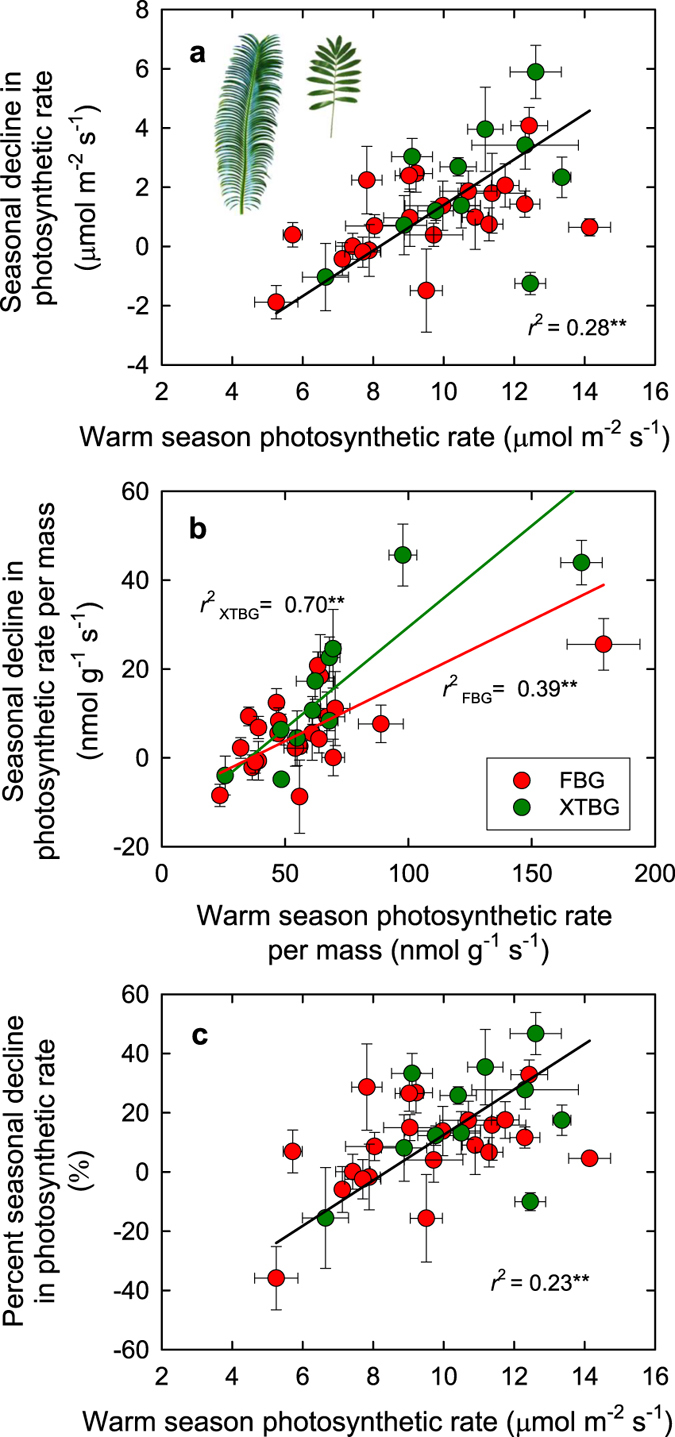
The relationship between maximum photosynthetic performance and seasonal maintenance into the cool season in cycads. **(a)** The relationships between seasonal decrease in net photosynthetic rate per unit leaf area (*A*_a_) and warm season *A*_a_ (**a**), between seasonal decrease in net photosynthetic rate per unit leaf mass (*A*_m_) and warm season *A*_m_ (**b**), and between percent seasonal decrease in photosynthetic rate (*A*) and warm season *A*_a_ (**c**). Points denote means ± SEs. Red points represent cycads in Fairylake Botanical Garden (FBG), while green points cycads in Xishuangbanna Tropical Botanical Garden (XTBG). The black lines are standardized major axis (SMA) lines fitted to all the points. The red line is a SMA line fitted to the species in FBG, while green line species in XTBG. *P < 0.05; **P < 0.01; ***P < 0.001. Photo credit: Y-J Zhang.

**Figure 3:**
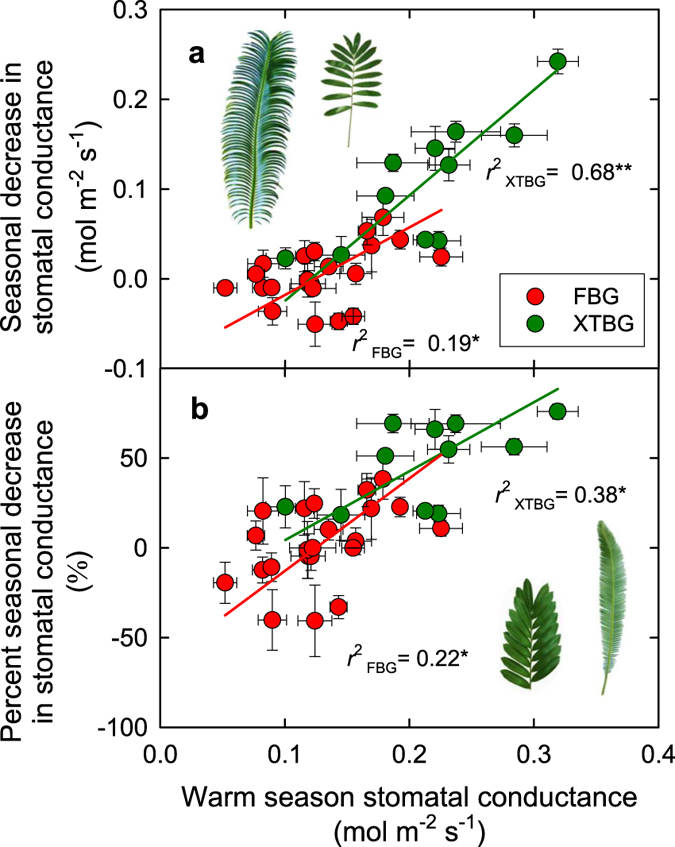
The relationships between seasonal decrease in stomatal conductance (*g*_s_) and *g*_s_ (**a**), and between percent seasonal decrease in *g*_s_ and warm season *g*_s_ (**b**). Points denote means ± SEs. Red points represent cycads in Fairylake Botanical Garden (FBG), while green points cycads in Xishuangbanna Tropical Botanical Garden (XTBG). The red lines are standardized major axis (SMA) lines fitted to the species in FBG, while green lines species in XTBG. *P < 0.05; **P < 0.01; ***P < 0.001. Photo credit: Y-J Zhang.

The hypothesized tradeoff between maximum photosynthetic rate in the favorable season and the ability to maintain photosynthesis through the unfavorable season was further confirmed in the experimental study across cycad species. Like many tropical and subtropical species that are chilling sensitive^2^,^4^, even when well-watered, cycads at our study sites show seasonal declines in leaf light-saturated net photosynthetic rate per leaf area or mass (*A*_a_, *A*_m_) and stomatal conductance (*g*_s_) in the cool season relative to the warm season (Fig. S2, S3), with strong species variation within each season and in the responses to season (*P* < 0.001 for season and species effects, and *P* < 0.001 for the species × season interaction; repeated-measures ANOVA). The decline of *A*_a_ in the cool season was positively related with the *A*_a_ measured during the warm season (Fig. 2a); species with higher photosynthesis showed stronger seasonal lows in the cool season. This positive relationship was stronger when photosynthetic rate was expressed per unit leaf mass (Fig. 2b). The decrease in *A* expressed in relative terms (*i.e.*, the % decline) was also positively related to *A*_a_ measured during the warm season (Fig. 2c). Similarly, the seasonal decline of stomatal conductance (*g*_s_) in cycads was associated with the warm season *g*_s_ across species (Fig. 3). Cycads from XTBG and FBG followed the same general trend, and because of the higher warm season *g*_s_ and higher seasonal decline of *g*_s_ in XTBG than in FBG (Fig. S4), there were significant differences in slope (Fig. 3a) or intercept (Fig. 3b) of the relationships among these variables between the two study sites (see additional discussion on differences between two sites in the Supplementary Information).

Thus, our study provides strong evidence for a general tradeoff across species between light-saturated photosynthetic rate in the favorable season, and the ability to maintain photosynthesis in the relatively unfavorable season. This tradeoff implies a constraint in leaf physiology that is consistent in its logic and potentially in mechanistic basis with the well-recognized tradeoff observed across species between maximum photosynthetic rate and leaf lifespan^9^,^10^,^11^,^12^. Moreover, this tradeoff is a factor that would contribute to diversification of maximum photosynthetic rates across species, such that species with lower rates can compensate to some degree with photosynthetic maintenance across seasons. This tradeoff was more pronounced in the meta-analysis than the common garden study, consistent with the greater variation in *A*_a_ and its seasonal decline across the phylogenetically diverse species of seed plants in the meta-analysis. Further, the tradeoff was stronger when expressed per unit leaf mass in the common garden study, consistent with the hypothesis that mass-based traits reflect leaf economics (*i.e.*, leaf construction cost and lifetime carbon return) more closely than area-based traits^22^. While our meta-analysis included only evergreen seed plants and all cycads are evergreen, the trade-off we highlighted would encompass plants of all leaf lifespans, including deciduous. Just as deciduous species tend on average to have higher photosynthetic rates during the growing season than evergreen species^23^, they have drastic declines in photosynthetic rates in the unfavorable season (to negligible levels, i.e., only that achieved by photosynthetic stems).

**Figure 4:**
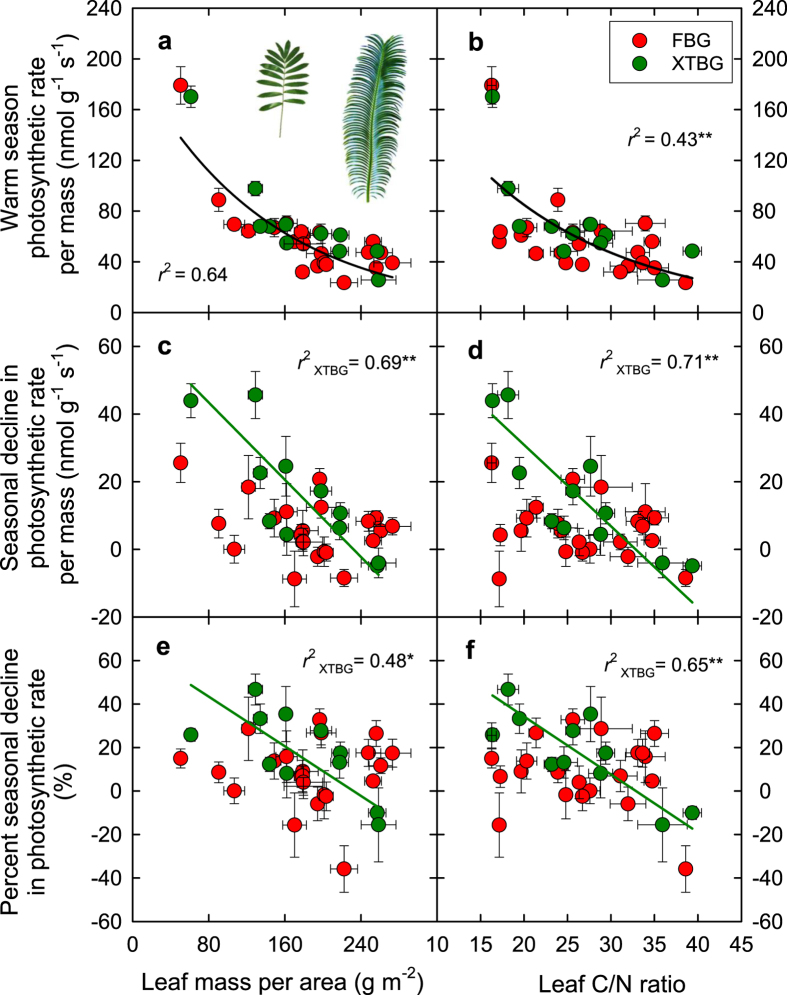
Warm season net photosynthetic rate per unit leaf mass (*A*_m_; **a,b**), seasonal decrease in *A*_m_ (**c,d**), and relative (percent) seasonal decrease in *A*_m_ (**e,f**) in relation to leaf mass per area (LMA) and leaf C/N ratio. Points denote means ± SEs. Red points represent cycads in Fairylake Botanical Garden (FBG), while green points cycads in Xishuangbanna Tropical Botanical Garden (XTBG).The black lines are exponential relationships (**a,b**) fitted to all the points. The green lines are standardized major axis lines (**c,d,e,f**) fitted to the species in XTBG. *P < 0.05; **P < 0.01. Caution is needed when interpreting the *r*^2^ value in (**a**) as *A*_m_ is calculated as photosynthetic rate per area (*A*_a_) divided by LMA, which raises the issue of non-independence. Photo credit: Y-J Zhang.

The mechanistic basis for this tradeoff may relate in part to leaf biomass investment per leaf area (LMA) and relative investment in construction and physiological function (C/N). High biomass investment in leaf thickness and/or compactness, and in high fiber, lignin, and structural components are associated with resistance to drought, freezing, and chilling^24^,^25^,^26^, but on the other hand this allocation of dry mass to structure “dilutes” (i.e., decreases the mass concentration of) the photosynthetic apparatus^10^ and increases the diffusion resistance of CO_2_ to the chloroplasts^11^,^15^,^16^,^17^,^18^, resulting in a low photosynthetic performance per unit leaf area and leaf mass. We found support for that hypothesis in our common study for cycads. High leaf mass per area (LMA) and C/N ratio in cycads were associated with low net photosynthetic rate per unit leaf mass (*A*_m_) (Fig. 4a,b), and also to lower absolute or relative seasonal declines in *A*_m_ (Fig. 4c–f). Notably, caution is needed when interpreting the relationship between *A*_m_ and LMA (Fig. 4a) as *A*_m_ is calculated as *A*_a_ divided by LMA, which raises the issue of non-independence. Yet, the LMA is expected to be one among a suite of traits involved in the balance between maximum photosynthetic rate and its maintenance in unfavorable conditions. While a higher LMA is associated with chilling resistance and low growing temperatures^25^,^27^, its direct or indirect mechanistic contribution to tolerance of photosynthesis to low temperature requires further study. In addition, this tradeoff could arise in part from variation in relative investment in biochemical chilling resistance. Chilling resistance is related to the desaturation degree of the membrane fatty acids^28^, high plasticity of photochemistry^29^, compatible solute concentration^30^, and proteins related to soluble sugar synthesis and antioxidant processes^31^, all of which may potentially decrease the nutrient investment in photosynthetic capacity and contribute to the formation of the trade-off between maximum performance and seasonal maintenance. Additionally, leaves with high photosynthetic capacity generally require high water transport to meet the associated high transpirational demand, which may be associated with greater vulnerability to loss of hydraulic conductance and larger declines in photosynthesis under adverse conditions^32^.

The tradeoff between maximum photosynthetic performance and the ability to maintain gas exchange in the unfavorable season in seed plants is consistent and extends cost-benefit theory as applied to plant physiology and ecology. This pattern extends the well-known tradeoff between maximum photosynthetic rate and leaf toughness/leaf lifespan to a different scale—that of leaf functional maintenance throughout the year. This trade-off implies a key constraint in leaf physiology, which would contribute to the diversification of maximum photosynthetic rates across species. These relationships can be used to generate hypotheses for seasonal patterns in photosynthesis based on a species’ maximum photosynthetic rate in the favorable season. For example, these relationships lead to the hypothesis that species with higher maximum photosynthetic rates would show stronger declines with stresses associated with ongoing climate change. Testing these implications and potential applications of this trade-off opens new avenues for further studies of the phenology of plant physiology in plants across lineages and ecosystems.

## Materials and Methods

### Analysis of published data to test for a tradeoff between maximum photosynthetic capacity and its seasonal maintenance in seed plants

We compiled a dataset for seasonal dynamics in light saturated photosynthetic CO_2_ assimilation (*A*_a_; μmol·m^–2^·s^–1^) of for evergreen seed plants including angiosperms and conifers from published studies of temperate sites around the world (see Table S1 in Supporting Information); data compilation followed Granda *et al*.^5^, which analyzed seasonal trends in daily stomatal conductance, chlorophyll fluorescence and stem xylem hydraulic conductance. For consistency, we selected only data for plants under natural conditions measured in the morning. Our compiled database included 62 data points for 36 species in given locations from 22 studies (see Table S1 in Supplementary Information). We distinguished sites with two types of major seasonal stresses (drought or low temperatures) that resulted in seasonal declines in *A*_a_. For the sites without summer drought, summer was defined as the favorable season and winter as the unfavorable season; *A*_a_ generally declined distinctly in the winter. For the sites with a pronounced summer dry season, and which generally had higher *A*_a_ in the winter than in summer, the wet season/winter was defined as the favorable season and the dry season/summer as the unfavorable season. We analyzed data for these two types of sites separately, and combined.

### Common garden study of cycads: study sites and plant material

The study was performed in two tropical botanical gardens in southern China with similar climates: Xishuangbanna Tropical Botanical Garden (XTBG; 21°41′N, 101°25′E, elevation 570 m) in Mengla, Yunnan Province, and Fairylake Botanical Garden (FBG; 22°34′N, 114°10′E, elevation 100–130 m) in Shenzhen, Guangdong Province. Mean annual temperature and mean annual precipitation at XTBG and FBG are 21.7 °C and 1560 mm, and 22.4 °C and 1933 mm, respectively (data from Xishuangbanna Station for Tropical Rain Forest Ecosystem Studies and Shenzhen Weather Station). At both sites about 80% of the rainfall occurs during the warm rainy season in May–October, with a dry, cool season in November–April (Fig. S1), during which the average daily minimum temperature is approximately 14 °C (Fig. S1). Ambient maximum photosynthetic photon flux density on sunny days (PPFD) in warm and cool season is around 2200 and 1650 μmol m^−2^s^−1^, respectively. Total daily net radiation in warm and cool season is 30 and 32 mol m^−2^ day^−1^, respectively, and lower in warm season due to more rain events. The soil of the Cycad Garden at XTBG is sandy alluvium and the soil of the National Cycad Germplasm Conservation Center at FBG is yellow soil^33^,^34^. Plants were kept well supplied with water throughout the year by frequent irrigation. We conducted the physiological measurements at XTBG in the cool season and warm season of 2009, and at FBG in the warm season of 2011 and the cool season of 2012. While the time elapsed between seasons results in uncertainty in resolving the effects of seasonality relative to aging, given the long leaf lifespan of cycads^35^,^36^, the effect of leaf aging on photosynthetic rate was expected to be minor relative to seasonal influences. Further, conducting measurements in the cool season followed by warm season at FBG, but in the warm season followed by cool season at XTBG, was expected to highlight any existing aging effects.

A common garden approach with plant materials from different habitats allows assessment of trait correlations controlled by genetic factors^37^. We selected 29 cycad species from seven genera within two families for the common garden study. Ten species were in the genus *Cycas* (family Cycadaceae), and 19 species in the genera *Ceratozamia, Dioon, Encephalartos, Lepidozamia, Macrozamia*, and *Zamia* (family Zamiaceae) (Table S2). All common garden species studied were in sun-exposed environments, and shaded plants (cycad species that do not grow well in sun-exposed habitats) were not selected for the present study.

### Common garden study of cycads: measurements of leaf physiology and structure

Previously published values for warm season *A*_a_, *A*_m_, *g*_s_, and LMA of the cycad species^19^ were combined with cool season measurements for this study. Sun-exposed healthy and mature individuals were used for physiological measurements. Six mature leaves (at least one-year-old) from three to six individuals per species were selected for the measurements, with the same individuals sampled for warm and cool season measurements. We aimed at characterizing leaf level performance and leaf design principles, and therefore individual leaves were treated as replicates for the physiological measurements and the statistical analyses. For each leaf, one leaflet was measured; leaflets were considered as the unit for measurement, being developmentally and functionally analogous to simple leaves or leaflets of angiosperms, typically measured in studies of photosynthetic rate.

We measured light-saturated net leaf photosynthetic rate per area (*A*_a_), stomatal conductance (*g*_s_), and intercellular CO_2_ concentration (*C*_i_) using a portable photosynthesis system (LI-6400, LI-COR, Nebraska, USA). Leaves were measured on sunny days between 0830 and 1030 h, at a photosynthetic photon flux density of 1500 μmol m^−2^ s^−1^, and at ambient temperature, humidity and CO_2_ concentration. We measured gas exchange at ambient rather than controlled temperatures to assess actual net photosynthetic performance for different seasons and its seasonal decline as a test for a general tradeoff, including the effects of cold acclimation rather than distinguishing instantaneous temperature effects. Average relative humidity during the measurement was 66.8 and 55.7% in the warm season at XTBG and FBG, respectively, and 68.7 and 66.4% respectively during the cool season, remaining high due to occasional rain and frequent fogs. Average vapor pressure deficit during the measurement was 1.58 and 1.95 kPa in the warm season at XTBG and FBG, respectively, and 1.08 and 1.48 kPa respectively during the cool season. Average air temperature during the measurement was 32.7 and 29.5 °C in the warm season at XTBG and FBG, respectively and 24.4 and 28.6 °C respectively during the cool season. Air temperatures remained high in sunny mornings of the cool season, and night temperatures were substantially lower in the cool season compared to the warm season (≈14 °C versus ≈24 °C) for both sites.

Net photosynthetic rate per mass (*A*_m_) was determined as *A*_a_ divided by leaf dry mass per unit area (LMA). For determination of LMA, we measured leaf area (using a LI-3000A area meter; LI-COR, Nebraska, USA), and weighed the leaves after oven-drying at 70 °C to constant mass. Leaf C and N concentrations were determined using a Vario MAX CN auto element analyzer (Elementar Analysensysteme, Germany) after oven-drying leaves at 70 °C for 48 h, and C:N ratios were calculated as the quotient.

### Data analysis

Data compiled from published data for seed plants were analyzed with standardized major axis (SMA) tests using SMATR V2.0^38^ to test the relationship between maximum *A*_a_ and absolute or percent declines of *A*_a_ in the unfavorable season (winter or dry season). Data were analyzed for two types of sites (sites with drought or low temperatures as the major seasonal stress) combined, and for each type of site separately (see the first section of Materials and Methods).

Data from the cycad common garden experiment were analyzed using paired t-tests and repeated measures analysis of variance (ANOVA). The seasonal differences in *A*_a_, *A*_m_, *g*_s_, and *C*_i_ for each given species were tested using paired *t*-tests applied to the leaf-level measurements for each species. Additionally, a repeated-measures analysis of variance (ANOVA) was used to test *A*_a_, *A*_m_, *g*_s_, and *C*_i_ for the overall effects of species, season, garden and their interactions, with season as the repeated factor. Standardized major axis (SMA) tests were used to test the relationships between *A (A*_a_ and *A*_m_) or *g*_s_ measured in the warm season and their declines in the cool season. One-way ANOVAs were also used to test for overall differences in average *A*_a_, *g*_s_, *A*_m_, and LMA between the two gardens^39^. Additionally, we tested for differences between the two gardens in the bivariate relationships among variables. For each relationship we tested for differences in slope between the two gardens using SMA tests using SMATR V2.0^38^; intercept (elevation) tests were performed if slopes were similar. If there were no significant differences, the data for both gardens were pooled and one common line was fitted to the data. Although % decline in *A* was calculated using the favorable season *A*, and *A*_m_ was calculated from *A*_a_ and LMA, both % decline in *A* and *A*_*m*_ have independent and important biological meanings (for detailed discussion, see Westoby *et al*.^22^). Therefore, the relationships between % decline in *A* and favorable season *A*, and between *A*_m_ and LMA were presented. Statistical analyses were performed using SPSS V21 (IBM Corp., Armonk, NY, USA) and Minitab V16 (Minitab, Inc., State College, PA, USA).

## Additional Information

**How to cite this article**: Zhang, Y.-J. *et al*. Speed versus endurance tradeoff in plants: Leaves with higher photosynthetic rates show stronger seasonal declines. *Sci. Rep.* **7**, 42085; doi: 10.1038/srep42085 (2017).

**Publisher's note:** Springer Nature remains neutral with regard to jurisdictional claims in published maps and institutional affiliations.

## Supplementary Material

Supplementary Information
