# Supplementary material for: Speed versus endurance tradeoff in plants: Leaves with higher photosynthetic rates show stronger seasonal declines
Source: Sci Rep. 2017 Feb 10;7:42085. doi: 10.1038/srep42085 (PMC5301202; doi:10.1038/srep42085)
Supplement: Supplementary Information [file srep42085-s1.pdf]

Supplementary Information for:

Speed versus endurance tradeoff in plants: Leaves with higher photosynthetic rates show stronger seasonal declines

Yong-Jiang Zhang, Lawren Sack, Kun-Fang Cao, Xue-Mei Wei & Nan Li

Tables S1, S2, Figures S1, S2, S3, S4, Additional discussion on differences between two gardens, Data source references

Table S1 Species, geographic location and the data source for the meta-analysis of the tradeoff between photosynthetic capacity (A_a, μmol CO_2 m^{-2} s^{-1}) and the maintenance of A_a into the unfavorable season

Species	Geographic location	Data source
**Sites without summer drought**		
Evergreen angiosperms		
Camellia japonica	Kamigamo, Japan	Miyazawa and Kikuzawa (2005)
Castanopsis cuspidata	Kamigamo, Japan	Miyazawa and Kikuzawa (2005)
Cleyera japonica	Kamigamo, Japan	Miyazawa and Kikuzawa (2005)
Ilex pedunculosa	Kamigamo, Japan	Miyazawa and Kikuzawa (2005)
Pachysandra terminalis	Hokkaido, Japan	Yoshie and Kawano (1986)
Photinia glabra	Kamigamo, Japan	Miyazawa and Kikuzawa (2005)
Quercus glauca	Kamigamo, Japan	Miyazawa and Kikuzawa (2005)
Conifers		
Picea abies	Lägeren, Switzerland	Falge et al. (1996)
Picea glauca	British Columbia, Canada	Binder and Fielder (1996)
Pinus sylvestris	Västoman, Sweden	Strand et al. (2002)
Pinus taeda	North Carolina, USA	Ellsworth (2000)
Pinus taeda	North Carolina, USA	Murthy et al. (1997)
Pinus taeda	Louisiana, USA	Tang et al. (2003)
**Sites with summer drought**		
Evergreen angiosperms		
Arbutus unedo	Mallorca, Spain	Gulías et al. (2009)
Boscia albitrunca	Oena Mine, South Africa	Wand et al., 1999
Citisus albidus	SaVall, Mallorca, Spain	Gulías et al. (2009)
Citisus albidus	Lloret, Mallorca, Spain	Gulías et al. (2009)
Citisus albidus	Puigpunyent, Mallorca, Spain	Gulías et al. (2009)
Citisus monspeliensis	SaVall, Mallorca, Spain	Gulías et al. (2009)
Citisus monspeliensis	Lloret, Mallorca, Spain	Gulías et al. (2009)
Citisus monspeliensis	Puigpunyent, Mallorca, Spain	Gulías et al. (2009)

Citisus monspeliensis	Binifaldó, Mallorca, Spain	Gulías et al. (2009)
Citisus salvifolius	Puigpunyent, Mallorca, Spain	Gulías et al. (2009)
Cneorum tricoccon	Binifaldó, Mallorca, Spain	Gulías et al. (2009)
Erica multiflora	Barcelona, Spain	Prieto et al. (2009)
Erica multiflora	Barcelona, Spain	Llorens et al. (2003)
Euclea pseudebenus	Oena Mine, South Africa	Wand et al. (1999)
Globularia alypum	Barcelona, Spain	Prieto et al. (2009)
Globularia alypum	Barcelona, Spain	Llorens et al. (2003)
Hypericum balearicum	Binifaldó, Mallorca, Spain	Gulías et al. (2009)
Olea europaea	SaVall, Mallorca, Spain	Gulías et al. (2009)
Olea europaea	Lloret, Mallorca, Spain	Gulías et al. (2009)
Olea europaea	Puigpunyent, Mallorca, Spain	Gulías et al. (2009)
Olea europaea	Binifaldó, Mallorca, Spain	Gulías et al. (2009)
Olea europaea	Sfax, Tunisia	Ben Ahmed et al. (2007)
Phillyrea latifolia	Tarragona, Spain	Ogaya and Penuelas (2003)
Phillyrea latifolia	Binifaldó, Mallorca, Spain	Gulías et al. (2009)
Pistacia lentiscus	SaVall, Mallorca, Spain	Gulías et al. (2009)
Pistacia lentiscus	Lloret, Mallorca, Spain	Gulías et al. (2009)
Pistacia lentiscus	Puigpunyent, Mallorca, Spain	Gulías et al. (2009)
Pistacia lentiscus	Binifaldó, Mallorca, Spain	Gulías et al. (2009)
Pistacia lentiscus	Binifaldó, Mallorca, Spain	Flexas et al. (2001)
Pistacia lentiscus	SaVall, Mallorca, Spain	Flexas et al. (2001)
Quercus coccifera	Puigpunyent, Mallorca, Spain	Gulías et al. (2009)
Quercus coccifera	Teruel, Spain	Baquedano and Castillo (2007)
Quercus ilex	Puigpunyent, Mallorca, Spain	Gulías et al. (2009)
Quercus ilex	Binifaldó, Mallorca, Spain	Gulías et al. (2009)
Quercus ilex	Tarragona, Spain	Ogaya and Penuelas (2003)
Quercus ilex	Teruel, Spain	Baquedano and Castillo (2007)
Rhamnus alaternus	Lloret, Mallorca, Spain	Gulías et al. (2009)
Rhamnus alaternus	Puigpunyent, Mallorca, Spain	Gulías et al. (2009)
Rhamnus ludovici-salvatoris	Binifaldó, Mallorca, Spain	Gulías et al. (2009)
Schotia afra	Oena Mine, South Africa	Wand et al. (1999)

Tamarix usneoides	Oena Mine, South Africa	Wand et al. (1999)
Conifers		
Juniperus phoenicea	Teruel, Spain	Baquedano and Castillo (2007)
Juniperus thurifera	Guadalajara, Spain	Gimeno et al. (2012)
Pinus canariensis	Tenerife, Spain	Peters et al. (2008)
Pinus halepensis	Barcelona, Spain	Prieto et al. (2009)
Pinus halepensis	Hebron, Israel	Maseyk et al. (2008)
Pinus halepensis	Teruel, Spain	Baquedano and Castillo (2007)
Pinus palustris	Georgia, USA	Addington et al. (2004)
Pinus pinea	Northern Plateau, Spain	Pardos et al. (2010)

Table S2 Cycad species studied in Xishuganbanna Tropical Botanical Garden (XTBG) and Fairylake Botanical Garden (FBG), origin and native habitats. Nomenclature, distribution and native habitats were taken from flora of China (http://www.efloras.org/), PlantNet (The Plant Information Network System of the Botanic Gardens Trust; http://plantnet.rbgsyd.nsw.gov.au/), and PACSOA (Palm and Cycad Societies of Australia; http://www.pacsoa.org.au/)

Species	Family	Code	Original distribution	Native habitat
**XTBG**				
Cycas micholitzii Thiselton-Dyer.	Cycadaceae	CM	China, Vietnam, Laos	Low, scrubby woodland with substantial rainfall, seasonal dry broadleaf forests at the bottom of limestone mountains, semi-shaded thickets
C. panzhihuaensis L. Zhou &S. Y. Yang	Cycadaceae	CPa	China	Valley savannas, dry closed low woodlands or shrublands
C. parvula S. L. Yang ex D. Y. Wang	Cycadaceae	CP	China	Valley savannas, broadleaf forests in the valleys, shrublands on slopes
C. sexseminifera F. N, Wei	Cycadaceae	CSe	China, Vietnam	Rocky crevices in broad-leaved forests on limestone mountains
C. siamensis Miq.	Cycadaceae	CSi	China, Vietnam, Thailand, Burma	Low open woodlands, low hills, seasonal rain forests, dry forests
C. szechuanensis W.C. Cheng & L.K. Fu	Cycadaceae	CS	China	Moist closed forest or woodlands, valley and near streams
Encephalartos cupidus R. A. Dyer	Zamiaceae	EC	South Africa	Steep slopes, rocky grassland and open forests
E. gratus Prain	Zamiaceae	EG	Mozambique, Malawi	Steep to precipitous slopes in gorges near streams
E. hildebrandtii A. Braun &Bauche	Zamiaceae	EH	Kenya, Tanzania	Seasonally dry savanna woodlands, sandy plains and rocky hill tops
Zamia fischeri Miq.	Zamiaceae	ZFi	Mexico	Understory of evergreen and cloud forests
Z. furfuracea L. f.	Zamiaceae	ZF	Mexico	Coastal savannas, coastal sand dunes and offshore islands, can grow down to water
**FBG**				
Cycas debaoensis Y. C. Zhong & C. J. Chen	Cycadaceae	CD	China	Moist mixed evergreen and deciduous woodlands on limestone slopes
C. elongata (Leandri) D. Y. Wang	Cycadaceae	CE	Vietnam, Laos	Forests to open shrublands on slopes, on dry gritty soil derived from coarse siliceous granites

Table S2 continued

Species	Family	Code	Original distribution	Native habitat
**FBG**				
C. fairylakea D. Y. Wang	Cycadaceae	CF	China	Moist mixed evergreen forests
C. panzhihuaensis L. Zhou & S. Y. Yang	Cycadaceae	CPa	China	Valley savannas, dry closed low woodlands or shrublands
C. szechuanensis W. C. Cheng & L. k. Fu	Cycadaceae	CS	China	Moist closed forests or woodlands, valley and near streams
C. thouarsii R. Br. ex Gaudich	Cycadaceae	CT	Madagascar, Tanzania, Mozambique	Moist forests behind beaches
Ceratozamia mexicana Brongn	Zamiaceae	CeM	Mexico	Wet tropical lowland rainforests to drier mountainous areas
Dioon edule Lindl.	Zamiaceae	DE	Mexico	Tropical dry forests
D. mejiae Dyer ex Eichler	Zamiaceae	DM	Honduras, Nicaragua	Dry rocky canyons
D. spinulosum Dyer ex Eichl.	Zamiaceae	DS	Mexico	Rocky, limestone evergreen forests
Encephalartos ferox Bertol. f.	Zamiaceae	EF	South Africa, Mozambique	Moist evergreen forest to arid dense shrublands
E. gratus Prain	Zamiaceae	EG	Mozambique, Malawi	Steep to precipitous slopes in gorges near streams
Lepidozamia hopei Regel	Zamiaceae	LH	Australia	In or near rainforests
L. peroffskyana Regel	Zamiaceae	LP	Australia	Wet sclerophyll forests or on rainforest margins, littoral rainforests or open scrubby forests
Macrozamia communis L. A. S. Johnson	Zamiaceae	MC	Australia	Wet to dry sclerophyll forests, mostly on old beach sands but also on shallow sandy or stony soils on ridges
M. lucida L. A. S. Johnson	Zamiaceae	ML	Australia	Coastal wet sclerophyll forests
Zamia amblyphyllidia D. W. Stev.	Zamiaceae	ZA	Jamaica, Cuba, Porto Rico, USA, Bahamas	Moist and warm tropical grasslands and open forests

Table S2 continued

Species	Family	Code	Original distribution	Native habitat
**FBG**				
Z. floridana A. DC.	Zamiaceae	ZFl	USA, Cuba, Porto Rico, Dominica	Open pinelands, hammocks, pine-oak woods, scrub forests and coastal woodlands, warm and moist open forests
Z. furfuracea L. f.	Zamiaceae	ZF	Mexico	Coastal savannas, coastal sand dunes and offshore islands
Z. integrifolia L. f.	Zamiaceae	ZI	Cuba, Porto Rico, Cayman Islands, USA	Dry pinelands or dry oak woods, usually in sand or marl (coral rock), also in hammocks
Z. splendens Schutzman	Zamiaceae	ZS	Mexico	Moist tropical rainforests
Z. vazquezii D. W. Stev., Sabato & De Luca	Zamiaceae	ZV	Mexico	Moist semi-evergreen forests to pine-oak forests

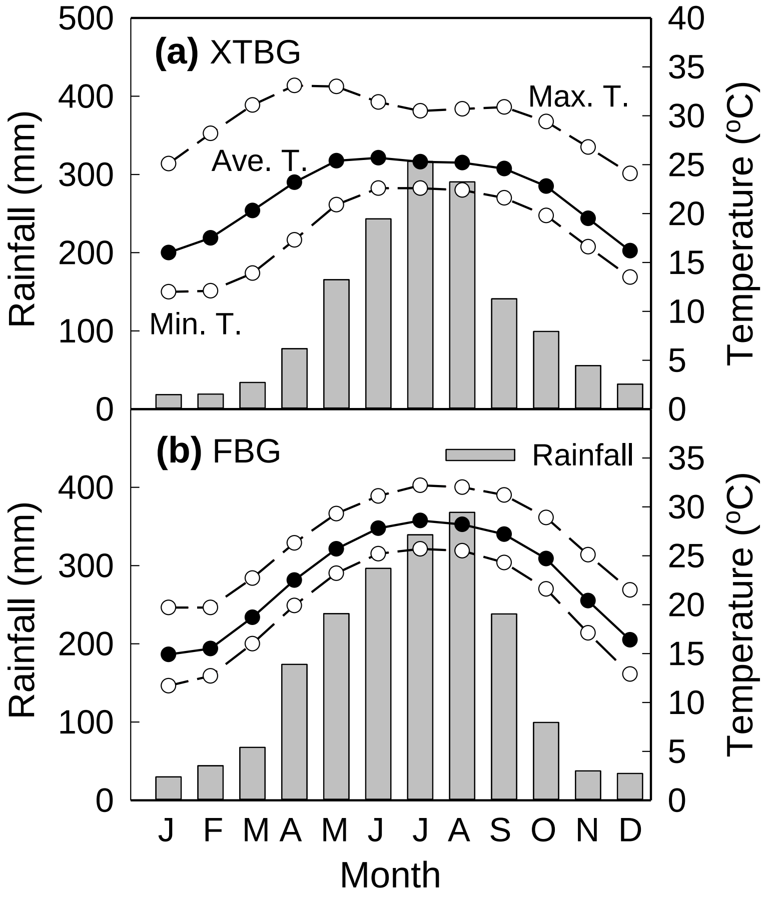

Figure S1. Mean monthly rainfall (bars), average maximum, minimum, and mean temperatures, in (a) Xishuangbanna Tropical Botanical Garden and (b) Fairylake Botanical Garden (Data from Xishuangbanna Tropical Rainforest Ecosystem Station and Shenzhen Weather Station).

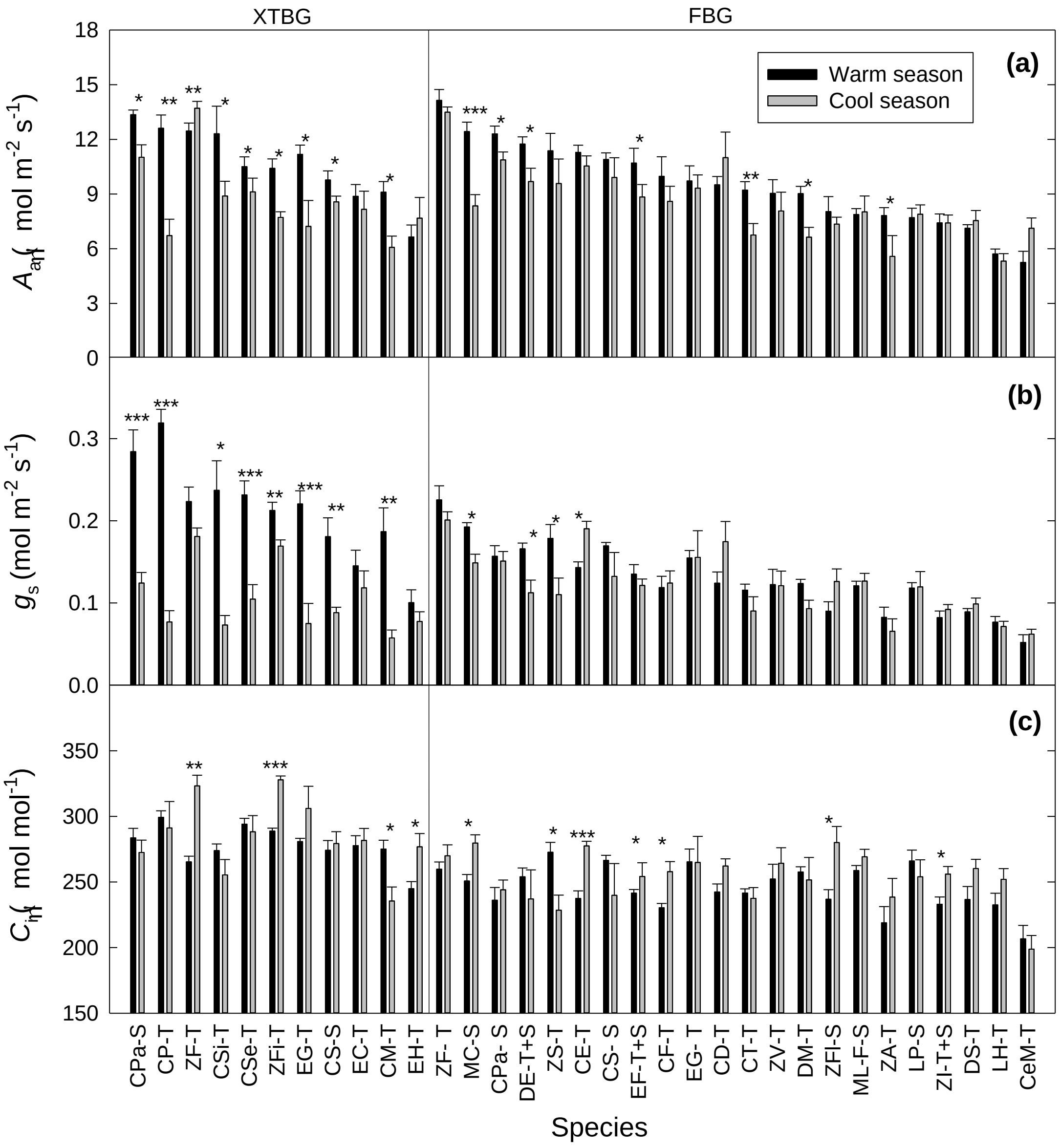

Figure S2. Net photosynthetic rates (A_a), stomatal conductance (g_s) and intercellular CO_2 concentration (C_i) of different cycad species in warm season and cool season. The letters followed species codes denote the distribution zone (T- Tropics; F- Subtropics). Bars are means + SE. * $P < 0.05$; ** $P < 0.01$; *** $P < 0.001$. Species codes are in Table S2.

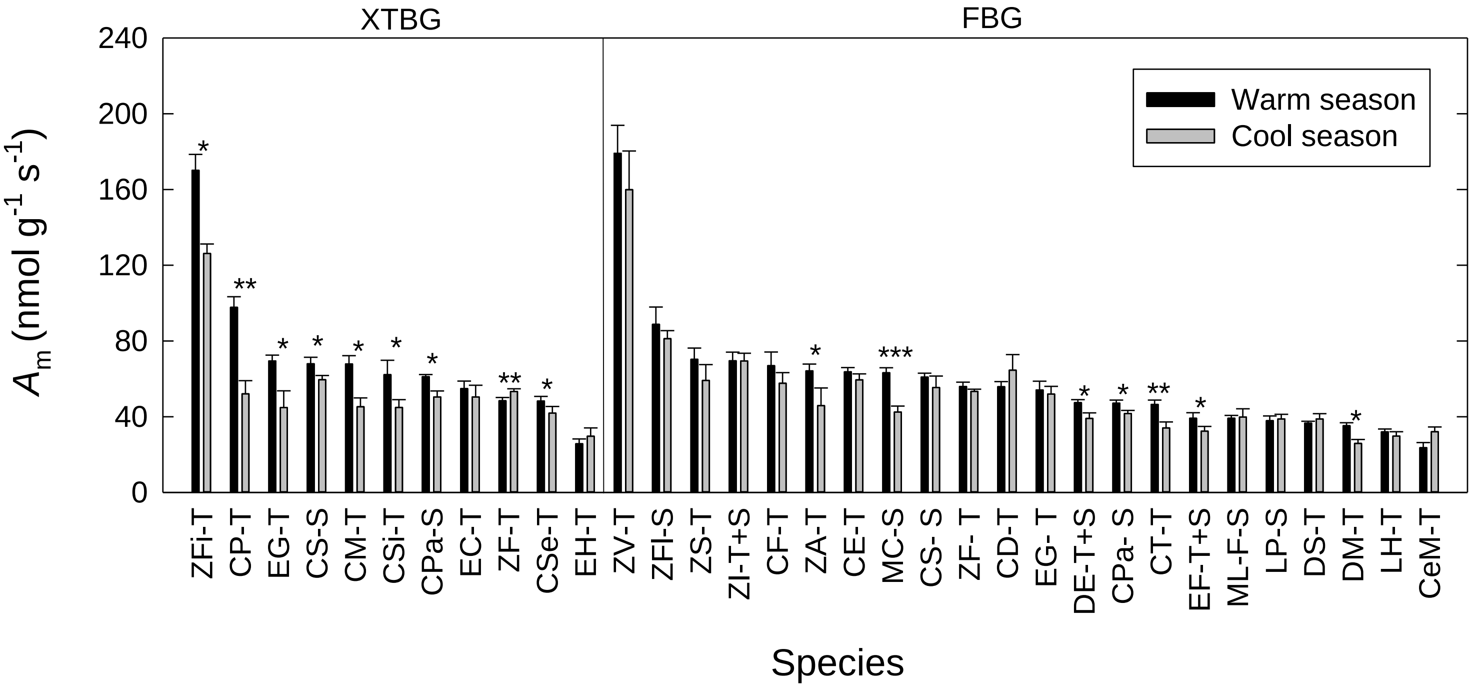

Figure S3 Light-saturated net photosynthetic rate per leaf dry mass (A_m) of different cycad species and averages of two different gardens in the warm season and cool season. The letters followed species codes denote the distribution zone (T- Tropics; F- Subtropics). Bars are Means + SEs. * $P < 0.05$; ** $P < 0.01$; *** $P < 0.001$. Species codes are in Table S2.

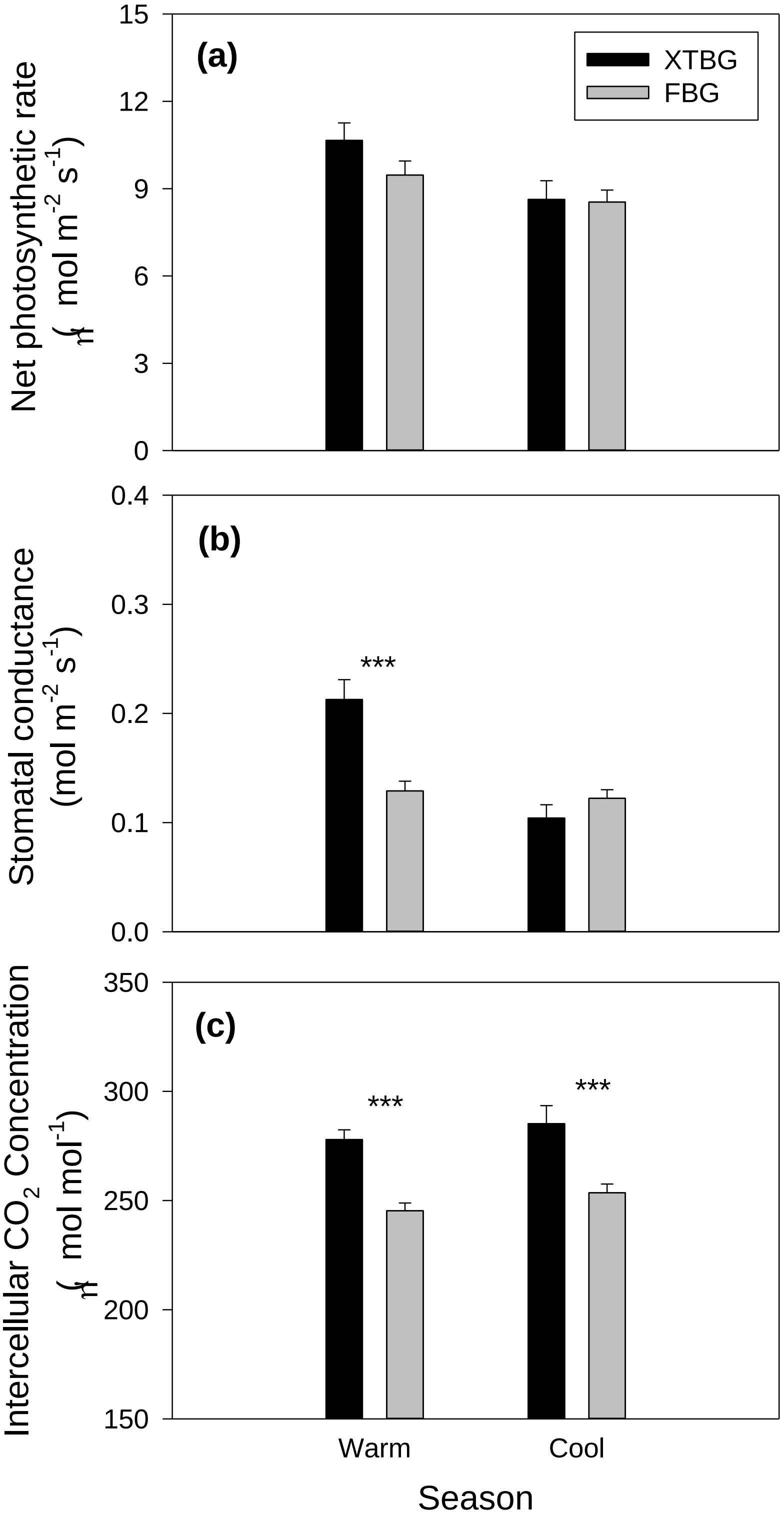

Figure S4 Differences in average light-saturated net photosynthetic rate per area, stomatal conductance, and intercellular CO_2 concentration between two different gardens in the warm and cool season. Means + SEs. *** indicate a significant difference between gardens ($P < 0.001$).

Additional discussion on differences between two gardens

The tradeoff between maximum performance and seasonal maintenance held across the two common garden study sites, but some trait relationships shifted. The difference in leaf stomatal conductance (g_s) and maximum photosynthetic rate per area (A_a) of the cycads between warm and cool seasons was smaller at FBG than XTBG (Fig. S2), despite no difference in cool season performance between the gardens (Fig. S4). Warm season photosynthesis was lower at FBG due to greater limitation by high Vapor pressure deficit (VPD; see *Materials and Methods*), as indicated by significantly lower intercellular CO2 concentrations (C_i) values (Fig. S4c). The greater seasonal changes in gas exchange for XTBG led to a tighter tradeoff between maximum values of A_a and g_s and their seasonal maintenance (Fig. 2b; Fig. 3a). This greater limitation of warm season gas exchange resulting in smaller range in seasonal changes in A likely impeded resolution of any linkage between seasonal decrease in maximum photosynthetic rate per mass (A_m) and leaf mass per area (LMA) or leaf C/N ratio in FBG.

Data source references

Addington, R. N., Mitchell, R. J., Oren, R. & Donovan, L. A. (2004) Stomatal sensitivity to vapor pressure deficit and its relationship to hydraulic conductance in Pinus palustris. *Tree Physiology,* **24,** 561-569.

Baquedano, F. J. & Castillo, F. J. (2007) Drought tolerance in the Mediterranean species Quercus coccifera, Quercus ilex, Pinus halepensis, and Juniperus phoenicea. *Photosynthetica,* **45,** 229-238.

Ben Ahmed, C., Ben Rouina, B. & Boukhris, M. (2007) Effects of water deficit on olive trees cv. Chemlali under field conditions in and region in Tunisia. *Scientia Horticulturae,* **113,** 267-277.

Binder, W. D. & Fielder, P. (1996) Seasonal changes in chlorophyll fluorescence of white spruce seedlings from different latitudes in relation to gas exchange and winter storability. *New Forests,* **11,** 207-232.

Ellsworth, D. S. (2000) Seasonal CO2 assimilation and stomatal limitations in a Pinus taeda canopy. *Tree Physiology,* **20,** 435-445.

Falge, E., Graber, W., Siegwolf, R. & Tenhunen, J. D. (1996) A model of the gas exchange response of Picea abies to habitat conditions. *Trees-Structure and Function,* **10,** 277-287.

Flexas, J., Gulias, J., Jonasson, S., Medrano, H. & Mus, M. (2001) Seasonal patterns and control of gas exchange in local populations of the Mediterranean evergreen shrub *Pistacia lentiscus* L. *Acta Oecologica,* **22,** 33-43.

Gimeno, T. E., Camarero, J. J., Granda, E., Pias, B. & Valladares, F. (2012) Enhanced growth of Juniperus thurifera under a warmer climate is explained by a positive carbon gain under cold and drought. *Tree Physiology,* **32,** 326-336.

Gulías, J., Cifre, J., Jonasson, S., Medrano, H. & Flexas, J. (2009) Seasonal and inter-annual variations of gas exchange in thirteen woody species along a climatic gradient in the Mediterranean island of Mallorca. *Flora,* **204,** 169-181.

Llorens, L., Penuelas, J. & Estiarte, M. (2003) Ecophysiological responses of two Mediterranean shrubs, Erica multiflora and Globularia alypum, to experimentally drier and warmer conditions. *Physiologia Plantarum,* **119,** 231-243.

Maseyk, K. S., Lin, T., Rotenberg, E., Gruenzweig, J. M., Schwartz, A. & Yakir, D. (2008) Physiology-phenology interactions in a productive semi-arid pine forest. *New Phytologist,* **178,** 603-616.

Miyazawa, Y. & Kikuzawa, K. (2005) Winter photosynthesis by saplings of evergreen broad-leaved trees in a deciduous temperate forest. *New Phytologist,* **165,** 857-866.

Murthy, R., Zarnoch, S. J. & Dougherty, P. M. (1997) Seasonal trends of light-saturated net photosynthesis and stomatal conductance of loblolly pine trees grown in contrasting environments of nutrition, water and carbon dioxide. *Plant Cell and Environment,* **20,** 558-568.

Ogaya, R. & Penuelas, J. (2003) Comparative seasonal gas exchange and chlorophyll fluorescence of two dominant woody species in a Holm Oak Forest. *Flora,* **198,** 132-141.

Pardos, M., Puertolas, J., Madrigal, G., Garriga, E., de Blas, S. & Calama, R. (2010) Seasonal changes in the physiological activity of regeneration under a natural light gradient in a Pinus pinea regular stand. *Forest Systems,* **19,** 367-380.

Peters, J., Gonzalez-Rodriguez, A. M., Jimenez, M. S., Morales, D. & Wieser, G. (2008) Influence of canopy position, needle age and season on the foliar gas exchange of Pinus canariensis. *European Journal of Forest Research,* **127,** 293-299.

Prieto, P., Penuelas, J., Llusia, J., Asensio, D. & Estiarte, M. (2009) Effects of long-term experimental night-time warming and drought on photosynthesis, Fv/Fm and stomatal

conductance in the dominant species of a Mediterranean shrubland. *Acta Physiologiae Plantarum,* **31,** 729-739.

Strand, M., Lundmark, T., Soderbergh, I. & Mellander, P. E. (2002) Impacts of seasonal air and soil temperatures on photosynthesis in Scots pine trees. *Tree Physiology,* **22,** 839-847.

Tang, Z. M., Chambers, J. L., Sword, M. A. & Barnett, J. P. (2003) Seasonal photosynthesis and water relations of juvenile loblolly pine relative to stand density and canopy position. *Trees-Structure and Function,* **17,** 424-430.

Wand, S. J. E., Esler, K. J., Rundel, P. W. & Sherwin, H. W. (1999) A preliminary study of the responsiveness to seasonal atmospheric and rainfall patterns of wash woodland species in the arid Richtersveld. *Plant Ecology,* **142,** 149-160.

Yoshie, F. & Kawano, S. (1986) Seasonal-Changes in Photosynthetic Characteristics of Pachysandra-Terminalis (Buxaceae), an Evergreen Woodland Chamaephyte, in the Cool Temperate Regions of Japan. *Oecologia,* **71,** 6-11.
